# Preserved reward outcome processing in ASD as revealed by event-related potentials

**DOI:** 10.1186/1866-1955-4-16

**Published:** 2012-05-31

**Authors:** James C McPartland, Michael J Crowley, Danielle R Perszyk, Cora E Mukerji, Adam J Naples, Jia Wu, Linda C Mayes

**Affiliations:** 1 Yale Child Study Center, Yale University School of Medicine, 230 South Frontage Road, New Haven, CT, USA

**Keywords:** Autism spectrum disorder, Reward processing, Event-related potentials, Electroencephalography, ERP, EEG, Feedback-related negativity, Medial-frontal negativity

## Abstract

**Background:**

Problems with reward system function have been posited as a primary difficulty in autism spectrum disorders. The current study examined an electrophysiological marker of feedback monitoring, the feedback-related negativity (FRN), during a monetary reward task. The study advanced prior understanding by focusing exclusively on a developmental sample, applying rigorous diagnostic characterization and introducing an experimental paradigm providing more subtly different feedback valence (reward versus non-reward instead of reward versus loss).

**Methods:**

Twenty-six children with autism spectrum disorder and 28 typically developing peers matched on age and full-scale IQ played a guessing game resulting in monetary gain (“win”) or neutral outcome (“draw”). ERP components marking early visual processing (N1, P2) and feedback appraisal (FRN) were contrasted between groups in each condition, and their relationships to behavioral measures of social function and dysfunction, social anxiety, and autism symptomatology were explored.

**Results:**

FRN was observed on draw trials relative to win trials. Consistent with prior research, children with ASD exhibited a FRN to suboptimal outcomes that was comparable to typical peers. ERP parameters were unrelated to behavioral measures.

**Conclusions:**

Results of the current study indicate typical patterns of feedback monitoring in the context of monetary reward in ASD. The study extends prior findings of normative feedback monitoring to a sample composed exclusively of children and demonstrates that, as in typical development, individuals with autism exhibit a FRN to suboptimal outcomes, irrespective of neutral or negative valence. Results do not support a pervasive problem with reward system function in ASD, instead suggesting any dysfunction lies in more specific domains, such as social perception, or in response to particular feedback-monitoring contexts, such as self-evaluation of one’s errors.

## Background

Difficulty with social interaction is a unifying feature of autism spectrum disorder (ASD), and reduced attention to social stimuli is evident early in development. Children with ASD demonstrate reduced sensitivity to biological motion [1] and orient less frequently to naturally occurring social stimuli relative to typically developing (TD) peers [2]. This primary reduction in attention to social stimuli has been hypothesized to stem from disruption of brain systems for assigning reward to social stimuli [3-7]. According to the social motivation model, atypical social attention reflects dysregulation of motivational mechanisms that, in typical development, direct an infant’s attention to socially relevant percepts [8]. Consequently, the child is deprived of essential social inputs during sensitive periods, disrupting subsequent development of social brain functions and associated behaviors [3,7]. In keeping with this suggestion, a number of studies have investigated the neural bases of reward processing in ASD and the specificity of atypical reward processing to social information in ASD. Behavioral studies indicate impaired generalization and inhibition of abstract stimulus-reward associations in ASD, suggesting that ventromedial prefrontal cortex dysfunction may contribute to impairment in responding flexibly to the unpredictable and nuanced nature of social reward [4]. Neuroimaging studies of reward processing in ASD have produced inconsistent results, indicating atypical patterns of reward circuitry activation in ASD but failing to establish whether reward-processing deficits in autism are specific to social stimuli or represent more general impairment. For example, Scott-Van Zeeland and colleagues reported attenuated ventral striatal response in ASD during social, but not monetary, reward learning, suggesting social reward-specific processing impairment; this response was not, however, associated with presumed behavioral measures of social reward (e.g., social reciprocity as measured by the Social Responsiveness Scale; SRS) [9]. In contrast to these findings of social-specific reward dysfunction, Dichter and colleagues demonstrated nucleus accumbens hypoactivation in individuals with ASD relative to TD individuals during both reward anticipation and outcome for monetary rewards, suggesting general reward-processing deficits in ASD [10]. While brain-imaging research has yet to clarify the nature of reward-processing difficulties in ASD, such studies have provided preliminary evidence of a role for reward circuitry dysfunction in the neuropathology of ASD [11].

The millisecond resolution of event-related potentials (ERPs) has been effective in revealing the temporal dynamics of reward processing [12]. Studies using ERPs offer the opportunity to measure neural responses at distinct processing stages representing specific mental events. In this way, they can individuate the components of a cognitive process, providing a nuanced method for examining the relationships between cognitive phenomena and behavior, such as the correlation between a given stage of reward processing and social function. Electrophysiological brain research has been critical in clarifying reward processes related to the evaluation of one’s reward-seeking behavior in response to feedback. The current study examined the feedback-related negativity (FRN), a negative-going deflection observed over frontocentral scalp approximately 250 ms after receiving feedback regarding an outcome that is worse than expected [13,14]. The FRN has been observed to reflect the valence of outcomes [15] and the magnitude of violations in probability expectations of an outcome [16]. Neural generators of the FRN have been localized to the medial-frontal cortex, including the anterior cingulate cortex (ACC) [13,17]. The FRN is presumed to reflect activity in the mesencephalic dopamine system, supporting feedback learning via transmission of reinforcement signals to the ACC that indicate errors in reward prediction [18,19]. In turn, the ACC is involved in integrating reward and loss valences, magnitudes, and probabilities to select and reinforce adaptive responses [20,21]. Neurobiological evidence of dysregulated dopamine metabolism [22-24] and abnormalities in structure [25], connectivity [26], and function [9,11] in the ACC in autism suggest disruption of this critical reward-feedback system may contribute to ASD symptomatology. The FRN may therefore serve as a useful metric of relative function/dysfunction at the feedback appraisal stage of reward processing in ASD, indexing difficulties in feedback integration and initiation of adaptive response. Two prior studies have attempted to examine the FRN in ASD. The first study, conducted by Groen and colleagues, examined ERPs during a feedback-learning task utilizing positive and negative feedback (i.e., win or lose points that could be later redeemed for a toy based on one’s performance on a given trial) in children with ASD, ADHD, and TD counterparts. Individuals with ASD did not differ from TD controls in early ERP components associated with feedback-outcome monitoring, but showed atypical ERP response during reward anticipation. The study had several notable limitations, including omission of gold-standard diagnostic procedures and use of a paradigm that failed to elicit a typical FRN in either group [27]. A second study by Larson and colleagues showed that, during a guessing task with monetary loss and gain feedback, children and adults with ASD ranging from 9 to 21 years of age demonstrated a robust FRN to loss relative to gain outcomes with amplitude comparable to TD peers. Neural response to reward feedback did not correlate with behavioral measures of inhibition, intelligence, anxiety, or symptom severity [19]. The authors interpreted these results as indicating that, under conditions of concrete, external feedback (versus more subtle internal feedback), individuals with ASD display typical reward-feedback appraisal.

The current study followed up on Larson and colleagues’ [18,19] suggestion that preserved FRN in ASD might reflect the concreteness of external feedback. We adapted their monetary reward-feedback paradigm to provide more subtle feedback regarding outcome, reducing the numeric quantity of gains and moving from gain versus loss to gain versus no gain (“draw”). Prior research in typical development indicates that all undesired outcomes (i.e., loss and draw) are processed equivalently [14]; however, the preservation of this binary evaluation system is unexplored in ASD. We also improved upon two limitations of prior studies on the FRN in ASD by adopting more rigorous gold standard research diagnostic criteria and restricting the age range to children. This latter adjustment is especially critical given developmental changes in the FRN [28-31] and reduced FRN associated with increased chronological age in ASD [19].

We evaluated two potential outcomes. First, replication of prior results of preserved reward-outcome processing (i.e., comparable FRN to draw trials) [19], would suggest normative feedback monitoring in ASD; as in typical development, the FRN in ASD may classify non-reward comparably to loss. This would add to the evidence for intact functioning of mechanisms subserving evaluation of external feedback regarding reward outcome in ASD. A second possible outcome, atypical neural response to non-reward cues in ASD (i.e., attenuated FRN to draw trials), would suggest that the quality of reward delivery differentially influences brain response in ASD; more ambiguous outcomes with subtler gradations of feedback may be processed differentially than in typical development (i.e., not in a binary fashion). This pattern of results would suggest a qualitatively distinct mechanism of reward-feedback monitoring in ASD. To examine the potential involvement of sensory mechanisms, we also compared groups on earlier temporal components reflecting more basic elements of visual perception, the N1 and P2 [32]. Finally, to explore relationships among behavioral characteristics and neural response to feedback cues, observed inconsistently in prior research, ERP parameters were correlated with behavioral measures of social function and dysfunction (Social Responsiveness Scale; SRS) [33,34], social anxiety (Social Anxiety Scale for Children; SASC-R) [35,36], and autism symptomatology indexed by both parent report (Autism Diagnostic Interview-Revised; ADI-R) [37] and clinical observation (Autism Diagnostic Observation Schedule; ADOS) [38].

## Methods

### Participants

Two groups participated in the study: children with ASD and medically and neuropsychiatrically healthy children with typical development (TD). Exclusionary criteria for participants with ASD included seizures, neurological disease, history of serious head injury, sensory or motor impairment that would impede completion of the study protocol, active psychiatric disorder (other than ASD; screened with the Child Symptom Inventory: 4th edition [39]), or anti-convulsant medications known to affect brain electrophysiology (alprazolam, clonazepam, diazepam, lorazepam, phenobarbital, and primidone). Additional exclusionary criteria for typical participants included the above plus learning/language disability or family history of ASD. All participants had normal or corrected-to-normal visual acuity. All procedures were conducted with the understanding and written consent of participants and their legal guardians and with approval of the Human Investigation Committee at the Yale School of Medicine.

All participants had Full Scale IQ scores in the typical range or higher (standard score of 70 or above on the Differential Ability Scales, 2nd edition [40] or the Wechsler Abbreviated Scale of Intelligence [41]). All children in the ASD group had a pre-existing diagnosis of ASD that was confirmed by gold standard diagnostic procedures: parent-interview (ADI-R) [37], semi-structured social behavior and communication assessment (ADOS) [38], and clinician diagnosis based on DSM-IV-TR criteria [42]. The ADI-R was not administered to two children because parents were unavailable to complete the interview. TD participants were recruited from an existing subject pool to match the ASD sample in terms of age, gender, ethnicity, handedness (Edinburgh Handedness Inventory [43]), and full-scale IQ. The final sample included 26 children with ASD and 28 typically developing children. TD and ASD participant characteristics are displayed in Table 1.

**Table 1:** Participant characteristics

	*N*	Sex	Age (years)		IQ		Handedness
		(*N*_male_)	*M* (*SD*)	Range	*M* (*SD*)	Range	(*N*_right_)
TD	28	17	12.08 (0.95)	10.13 - 13.50	109.61 (12.17)	87 - 135	23
ASD	26	22	11.22 (2.52)	7.78 - 15.02	103.77 (18.16)	72 - 133	23

### Behavioral measures

Additional questionnaire data were collected from the children in the ASD group. The Social Responsiveness Scale (SRS) is a 65-item questionnaire yielding continuous measures of social function and dysfunction [33,34]. In addition to providing an overall index of severity of social impairment, the SRS assesses social motivation, social cognition, social awareness, social expression, and autistic preoccupations. The parent report version of the SRS was used in the current study to measure the ability of children in the ASD group to engage in emotionally appropriate reciprocal social interactions. Prior work has found that SRS scores demonstrate the sensitivity to distinguish children diagnosed with a pervasive developmental disorder from children with other psychiatric disorders [33]. In addition, the SRS has demonstrated high internal consistency, test-retest reliability, inter-rater agreement, and concurrent validity with ADI-R algorithm scores [33,34]. The SRS was not collected from two participants because of lack of parent availability.

The subjective experience of social anxiety in children in the ASD group was measured using the Social Anxiety Scale for Children (SASC-R), an 18-item self-report questionnaire [35,36]. The SASC-R consists of three conceptually derived subscales assessing distinct aspects of the experience of social anxiety: (1) fear of negative evaluation by peers, (2) social avoidance and distress in new situations or with unfamiliar peers, and (3) more general or pervasive social distress, discomfort, and inhibition. High internal consistency, test-retest reliability, and concurrent validity with measures of social competence support the psychometric integrity of this measure in school-age children [35] and young adolescents [44]. SASC-R scores were not obtained from four participants in the ASD group because of lack of time during the experimental session. Scores on the ADOS, ADI-R, SRS, and SASC-R are reported in Table 2.

**Table 2:** Behavioral scores for the ASD group

	*M (SD)*	Range
ADOS total score	11.38 (3.60)	8–24
ADI Reciprocal Social Interaction total score	19.83 (5.27)	9–28
ADI Communication (Verbal) total score	15.50 (5.10)	8–24
SRS total score	78.75 (10.70)	52–90
SASC-R total score	42.78 (16.07)	22–86

### ERP procedures

#### *Task*

**Figure 1:**
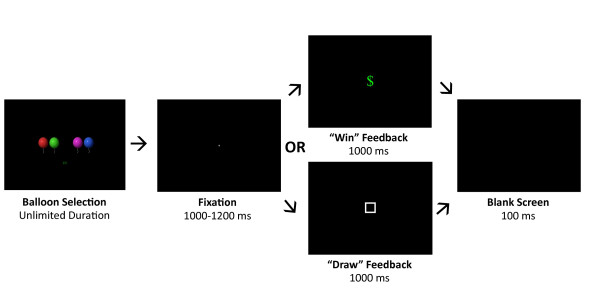
Trial sequence for win and draw conditions.

The task was a feedback-reward paradigm consisting of equiprobable “win” and “draw” outcomes across four blocks of 30 trials. Three additional winning trials were included such that the number of wins exceeded 50 percent; these were not included in ERP averages. Participants were shown four balloons of different colors (red, green, blue, and purple) on a computer screen and instructed to choose one of the balloons by pressing one of four corresponding buttons on a response box. They were informed that, if they chose the correct balloon, they would see a green dollar sign and win 10 cents (“win” condition). If they chose one of the “unlucky” balloons, they would see an empty white box and not win any money (“draw” condition). The paradigm was designed such that win and draw outcomes occurred in random order and a set number of times per block independent of the participant’s choice. Each trial consisted of: four balloons appearing on screen in a random order and remaining until the participant responded, a crosshair appearing for a duration randomly varying between 1,000-1,200 ms, either a green dollar sign or a white empty box appearing for 1,000 ms, and a blank screen appearing for 100 ms (see Figure 1). After each block of 30 trials, a screen appeared displaying a jar being filled with dimes to the sound of clinking coins. The jar progressively filled after each of the four blocks, culminating in a full jar by the end of the 10-min experiment, to maintain participant interest and motivation. Prior to beginning the game, there were three practice trials, which introduced the coin jar. All stimuli were presented in frontal view and at a standardized viewing size (10.6° by 8.1°) on a uniform black background.

#### *EEG data collection and processing*

Stimuli were presented on a 51-cm LCD monitor (60-Hz, 1,024 × 768 resolution) with E-Prime 2.0 software (Psychology Software Tools, Pittsburgh, PA [45]) at a viewing distance of 24 inches in a sound-attenuated room with low ambient illumination. A 128-lead Geodesic Sensor Net Hydrocel (Electrical Geodesics, Inc., Eugene, OR [46]) was fitted on the participant’s head according to the manufacturer’s specifications, and impedances were kept below 40 kilo-ohms. EEG was recorded continuously at 250 Hz (0.1 Hz highpass, 100 Hz lowpass) using NetStation 4.3. Cz served as the reference point for all electrodes.

EEG data were processed using NetStation v. 4.4 software. Data were low-pass filtered offline at 30 Hz prior to segmentation. Filtered data were then segmented to an epoch lasting from 100 ms before to 600 ms after stimulus onset. Artifact detection settings were set to 200 μv for bad channels, 140 μv for eye blinks, and 100 μv for eye movements. Channels with artifacts on more than 40% of trials were marked as bad channels and replaced through spline interpolation. Segments that contained eye blinks, eye movement, or more than ten bad channels were marked as bad and excluded. Automated artifact detection was confirmed via hand editing for each subject for each trial. Data were re-referenced to an average reference and baseline corrected to the 100-ms pre-stimulus epoch. Trial-by-trial data were subsequently averaged at each electrode for each condition, i.e., “win” and “draw,” separately for every individual. Participants with more than 75% (45) bad trials were excluded from analysis. For the TD group, an average of 35 artifact-free trials per participant was obtained in the win condition and an average of 29 was obtained in the draw condition. For the ASD group, an average of 36 artifact-free trials per participant was obtained in the win condition and an average of 29 was obtained in the draw condition; two-tailed t-tests showed no difference between groups for comparisons between win (*p* = 0.800) and draw (*p* = 0.978) conditions, respectively. Electrodes of interest were selected based on maximal observed amplitude of the FRN and on prior research [18,47-49]; amplitude and latency to peak for all ERP components (N1, P2, FRN) were extracted as the average across a cluster of four frontal electrodes (5, 6, 11, and 12) approximating Fz (mapping directly to electrode 11). Temporal windows for EEG components were based on inspection of the grand averaged waveforms and confirmed in individual averages. The N1 was measured as minimum amplitude within 50–150 ms from feedback onset, and the P2 was measured as the maximum amplitude within 150–250 ms from feedback onset. The FRN was measured as minimum amplitude in the window from 250–300 ms following feedback, as per previous work [50,51]. Additionally, we generated a difference wave for the win and draw conditions and estimated the peak amplitude of this difference wave in the 250–300 ms window. This approach was designed to control for possible confounding effects of earlier and later occurring electrophysiological components on the FRN [19,47]. Peak amplitude and latency for each component were exported to R and SPSS for analysis [52].

#### *Data analysis*

Amplitudes and latencies to peak for exogenous (N1, P2) and endogenous (FRN) components were analyzed separately using univariate repeated measures analyses of variance (ANOVA), with condition (win/draw) as the within-subjects factor and group as the between-subjects factor (ASD/typical). Separate independent sample t-tests were used for comparing difference wave amplitudes between groups, and Pearson correlations (Bonferroni corrected) were used for assessing the relationship between ERP components and behavioral measures. For all analyses, the significance level was set at α < 0.05.

## Results

**Figure 2:**
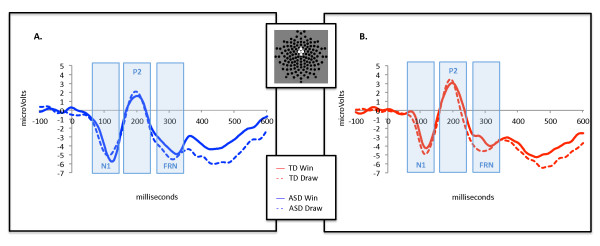
**Grand averaged waveforms elicited by “win” and “draw” events in children with ASD (*Panel A*) and typically developing controls (*Panel B*).***Highlighted* temporal windows indicate N1, P2, and FRN components.

Figure 2 displays grand averaged waveforms for both groups in the win and draw conditions.

### ERP measures

#### *N1 amplitude*

There were no significant effects of group or condition or interactions for N1 amplitude [all Fs < 3.8, all *p*s > 0.57, all η^2^_partial_s < 0.068]. Independent samples t-tests on the amplitude of the difference wave also revealed no differences between the two groups [t(52) = 0.889, *p* = 0.378].

#### *P2 amplitude*

There were no significant main effects or interactions for P2 amplitude [all Fs < 1.6, all *p*s > 0.22, all η^2^_partial_ s < 0.029]. Independent samples t-tests on the amplitude of the difference wave found no differences between the two groups [t(52) = 0.612, *p* = 0.542].

#### *FRN amplitude*

There was a significant main effect of condition [F(1, 52) = 5.65, *p* = 0.021, η^2^_partial_ = 0.098], revealing that the draw condition elicited significantly more negative amplitude than the win condition. There was no main effect of group [F(1, 52) = 0.197, *p* = 0.659, η^2^_partial_ = 0.002], nor was there a group by condition interaction [F(1, 52) = 0.128, *p* = 0.722, η^2^_partial_ = 0.004] that would suggest differences at the FRN between the individuals with ASD and TD peers. This finding was also supported by directly comparing the peak amplitude of the difference wave between individuals with ASD and controls, which did not reveal a significant difference [t(52) = 0.542, *p* = 0.59].

#### *Latency*

Prior research on the FRN has focused primarily on component amplitude rather than latency; however, given the relevance of latency to understanding social perception in ASD [53], we additionally conducted repeated measures ANOVA on latency to peak (minimum for N1, FRN; maximum for P2). There was a significant main effect of condition on N1 latency [F(1, 52) = 7.92, *p* = 0.007, η^2^_partial_ = 0.132], such that the draw conditions elicited an earlier N1 component than the win condition. However, there were no other significant effects of group or condition on component latency for either the P2 or the FRN.

#### *Behavioral measures*

To examine the relationship between brain activity and behavior in ASD, we computed difference scores between win and draw conditions for the FRN and explored their relationships with measures (total scores and subscores) of social anxiety (SASC-R), social function (SRS), and autism severity (ADI-R and ADOS). No significant correlations with any of the behavioral measures were detected [all *r*s < 0.37, all *p*s > 0.08]. Additional correlations were computed between FRN difference scores and age and IQ, and there were no significant associations [all *r*s < 0.09, all *p*s > 0.53].

## Discussion

The current study examined feedback outcome monitoring in the context of monetary reward in a rigorously characterized sample of children with ASD. To do so, we adapted an extant monetary reward-feedback paradigm [19] to provide more nuanced feedback regarding performance (gain versus no gain instead of gain versus loss), and we decreased the magnitude of gain. Despite these changes to the paradigm, we obtained results comparable to experiments using more salient feedback. Children with ASD showed neural response to feedback signals comparable to that of typically developing peers. These findings concord with a large body of work in typical adults suggesting that the FRN marks deviation from optimal outcome in a binary fashion and is insensitive to the relative magnitude of loss. Also, consistent with prior work examining response to feedback monitoring in ASD [19], we did not observe correlations between neural response and ASD symptomatology, cognitive ability, social skills, or social anxiety. In contrast to previous work, however, we did not observe a significant relationship between feedback monitoring and chronological age. This likely reflects our constrained age range; the previous study included adults and observed attenuated FRN in older individuals with ASD, who were not included in the current study.

Results of the current study add to the body of evidence suggesting normative feedback monitoring, as indexed by the FRN, in ASD. Several studies have, however, now demonstrated atypical neural response at an ERP index of internal monitoring of outcomes, the error-related negativity (ERN) in ASD [54-57]. In contrast to the FRN, which is elicited by external feedback regarding one’s performance, the ERN represents a rapidly occurring component elicited within approximately 100 ms of one’s own erroneous response. The ERN is considered to be a reflection of self-monitoring for errors or conflicts between actions consistent and inconsistent with desired end states. Despite their distinct functional conceptualizations, both components have been related to activity in the ACC [58-61]. In describing this inconsistency in reinforcement-signal-monitoring literature in ASD (intact FRN but atypical ERN), Larson and colleagues suggest that the relevant distinction in reward processing in ASD may be the salience of external relative to internal feedback, given the concrete cognitive style evidenced by individuals on the autism spectrum. In the current study, we observed a normative FRN in ASD, despite reduced feedback salience, suggesting that even with more subtle, nuanced external feedback, individuals with ASD display typical feedback monitoring. Our results indicate that prior findings of robust FRN response to loss extend to non-reward as well. Of course, the stimuli used in the current study remain dichotomous in nature, signaling gain and relative loss; feedback studies that contrast reward magnitude manifest in differential P300 effects rather than the FRN, which appears to be mainly sensitive to valence [62]. Future research using even more nuanced reward feedback (e.g., variable gains) might reveal differential neural response during feedback appraisal in ASD as a test of the hypothesis that preserved feedback monitoring is contingent upon the concreteness of feedback.

Our results inform understanding of the role of basic motivational deficits as the core dysfunction in ASD. Findings indicate normative monitoring of external feedback regarding reward outcomes. The observation of intact facets of reward circuitry in ASD indicates a complex role of motivation in the development of ASD. Our results are inconsistent with a model speculating pervasive dysfunction of reward circuitry in ASD. Indeed, given the specificity of the difficulties associated with the ASD phenotype (e.g., strong circumscribed interests despite reduced social interests), a primary, domain-general motivational impairment is unlikely. Instead, atypicalities in reward processing may be evident at specific processing stages or for particular kinds of information, possibilities not evaluated by this study. The current paradigm revealed intact function during monitoring of reward outcome, but it did not assess reward anticipation. Given prior evidence of atypical brain activity during reward anticipation despite normative function during reward outcome in ASD [27], “unpacking” reward system function in this way is a paramount goal for future research. The current study also focused exclusively on monetary reward. Given the pronounced social deficits that characterize ASD, others have suggested specific dysfunction in brain circuitry systems subserving social reward [63]. Studies to date have conceptualized social reward in several ways, including positive facial expressions, positive feedback, verbal praise, and altruistic ends [64,65]. Non-social rewards are most often money, food, or tangible rewards (e.g., toys). In ASD research, behavioral studies indicate diminished effects of social reinforcement in ASD relative to TD [66], and neuroimaging studies reveal selectively reduced activity in frontal-striatal reward circuitry to social reward in ASD compared to TD [9]. A single ERP study contrasting brain response to predictive cues of social and non-social reward [67] found general reward anticipation deficits at the P300 but intact reward outcome processing in ASD. Investigations of social versus non- social reward hold great promise to elucidate the idiosyncrasies of reward system function in ASD; however, several limitations of research to date can be improved in future research. The typical non-social reward, money, is not unambiguously non-social, as it may serve immediate social purposes for study participants (e.g., using the money to buy a videogame to play with siblings). Additionally, it is unclear to what extent typical experimental social rewards, such as static faces or dynamic video clips of smiling people, are truly rewarding. A challenge for our field is to develop increasingly ecologically valid assays to investigate the cognitive neuroscience of reward processing in ASD. As articulated above, we see this as involving (1) increasingly subtle gradations of reward feedback and (2) ecologically valid and construct valid social and non-social rewards. Work currently in progress in our laboratory attempts to address this issue through provision of simulated real-time feedback on one’s performance from a live observer.

An alternative possibility is that atypical patterns of reward processing in ASD may represent co-occurring features that moderate development rather than drive the primary deficits that characterize the disorder [56]. Such an idea is also consistent with the lack of specificity observed for abnormalities in reward processing systems, evident in individuals with subclinical mood symptoms [68,69], associated with variation in normative affective characteristics [70], and also apparent in other non-autistic psychiatric disorders [71,72]. Observed variability in feedback processing may represent a neuropsychological characteristic with great variability in individuals both on and off the autism spectrum that contributes to both typical and atypical development. Along with a variety of cognitive, behavioral, and affective factors, reward processing may therefore be instrumental in understanding heterogeneity in autistic development. Pending longitudinal research, atypicalities in reward system function in autism may also represent consequences of growing up with the disorder rather than core problems, per se.

Several limitations of the current study should be addressed in future work. Considering that our study used a variant of the experimental task employed in previous work demonstrating normative FRN response in ASD [19], it will be particularly important to demonstrate preserved FRN with other experimental paradigms. Though a robust FRN in both studies makes clear that the paradigm activated feedback-monitoring circuitry, more difficult or engaging tasks featuring subtler reward feedback might be required to elicit otherwise unobserved differences between individuals with ASD and TD. This notion is consistent with the observation that ERN paradigms eliciting differences in ASD have employed paradigms that were considerably more challenging. It should be noted, however, that the use of more complex experimental paradigms may necessitate greater cortical modulation of the basic reward processing mechanisms in question; given prior reports of atypical cortical connectivity in ASD, this may complicate interpretation of results [73,74]. Lastly, the absence of significant correlations between behavioral measures and ERP components in both current and prior FRN studies suggests that the reward-feedback mechanisms tapped by our study may be less relevant to autistic symptomatology or related neuropsychological and psychological characteristics than those employed in prior ERN studies.

## Conclusions

This study used a variant of an existing monetary reward-feedback paradigm to investigate feedback monitoring as indexed by the FRN. This was the first study to examine reward-feedback monitoring in a rigorously characterized developmental sample, and it was the first to contrast neural response to gain versus no gain rather than gain versus draw in ASD. Results of the current study were consistent with prior work demonstrating intact feedback monitoring. Children with ASD showed comparable brain activity to typical peers, and neural indices of feedback appraisal were unrelated to behavioral characteristics related to anxiety, autism symptomatology, or neuropsychological features. Our findings add to a body of evidence suggesting preservation of feedback monitoring mechanisms in ASD and further emphasize the need for ecologically valid studies to examine potential dysfunction within specific domains, such as social perception, and at discrete stages of reward processing.

## Competing interests

The authors have no financial or non-financial competing interests to declare.

## Authors’ contributions

JCM, MJC, and LCM conceptualized and designed the study. MJC and JW created the experimental paradigm. DRP, CEM, and MJC collected the data. CEM, DRP, and AJN processed and analyzed the data. All authors contributed to interpreting results and writing the manuscript. All authors read and approved the final manuscript.
